# Personalized Nutrition Strategies for Patients in the Intensive Care Unit: A Narrative Review on the Future of Critical Care Nutrition

**DOI:** 10.3390/nu17101659

**Published:** 2025-05-13

**Authors:** Mircea Stoian, Adina Andone, Sergiu Rareș Bândilă, Danusia Onișor, Dragoș-Florin Babă, Raluca Niculescu, Adina Stoian, Leonard Azamfirei

**Affiliations:** 1 Department of Anesthesiology and Intensive Care Medicine, George Emil Palade University of Medicine, Pharmacy, Sciences and Technology of Târgu Mureș, 540142 Târgu Mureș, Romania; mircea.stoian@umfst.ro (M.S.); leonard.azamfirei@umfst.ro (L.A.); 2 Intensive Care Unit, Mures Clinical County Hospital, 540103 Târgu Mureș, Romania; 3 Gastroenterology Department, George Emil Palade University of Medicine, Pharmacy, Sciences and Technology of Târgu Mureș, 540142 Târgu Mureș, Romania; adina.roman@umfst.ro (A.A.); danusia.onisor@umfst.ro (D.O.); 4 Orthopedic Surgery and Traumatology Service, Marina Baixa Hospital, Av. Alcade En Jaume Botella Mayor, 03570 Villajoyosa, Spain; bandila_ser@gva.es; 5 Department of Cell and Molecular Biology, George Emil Palade University of Medicine, Pharmacy, Sciences and Technology of Târgu Mureș, 540142 Târgu Mureș, Romania; dragos-florin.baba@umfst.ro; 6 Department of Pathophysiology, George Emil Palade University of Medicine, Pharmacy, Sciences and Technology of Târgu Mureș, 540136 Târgu Mureș, Romania; raluca.niculescu@umfst.ro

**Keywords:** enteral and parenteral nutrition, critical illness, metabolome, personalized nutrition, nutritional genomics, metabolic diseases

## Abstract

**Introduction:** Critically ill patients in intensive care units (ICUs) are at high risk of malnutrition, which can result in muscle atrophy, polyneuropathy, increased mortality, or prolonged hospitalizations with complications and higher costs during the recovery period. They often develop ICU-acquired weakness, exacerbated by sepsis, immobilization, and drug treatments, leading to rapid muscle mass loss and long-term complications. Studies indicate that adequate protein and calorie intake can decrease mortality and improve prognosis and recovery. However, optimal implementation remains a critical challenge. **Objectives:** This narrative review aims to summarize recent advances in nutritional strategies for critically ill patients. It highlights the benefits and limitations of current approaches including enteral (EN) and parenteral nutrition (PN) and examines their impact on clinical outcomes and overall mortality. Additionally, the review explores the emerging role of precision nutrition in critical care using technologies such as metabolomics and artificial intelligence (AI) to provide valuable insights into optimizing nutritional care in critically ill patients. **Methods:** A comprehensive literature search was conducted to identify recent studies, clinical guidelines, and expert consensus papers on nutritional support for ICU patients. The investigation focused on critical aspects such as the optimal timing for intervention, the route of administration, specific protein and energy targets, and technological innovations to support personalized nutrition, ensuring that each patient receives tailored support based on their unique needs. **Results:** Guidelines recommend initiating EN or PN nutrition within the first 48 h of admission, using indirect calorimetry (IC) to estimate energy needs, and supplementing protein up to 1.2 g/kg/day after stabilization. IC has gained importance in assessing energy needs but is still underused in the ICU. EN is preferred because it maintains intestinal integrity, reduces the risk of infections, and is recommended within the first 48 h of ICU admission. PN is used when EN is infeasible, but it increases the risk of infection. By integrating metabolomics with transcriptomic and genomic data, we can gain a deeper understanding of the effect of nutrition on cellular homeostasis, facilitating personalized treatments and enhancing the recovery of critically ill patients. **Conclusions:** AI is becoming increasingly important in monitoring and evaluating artificial nutrition, providing a more accurate and efficient alternative to traditional methods. AI can assist in identifying and managing malnutrition and is effective for estimating caloric and nutrient intake. AI minimizes human error, enables continuous monitoring, and integrates various data sources. The nutritional care of critically ill patients requires collaboration among specialists from diverse fields, including physicians, nutritionists, pharmacists, radiologists, IT experts, and policymakers.

## 1. Introduction

Some studies estimate that malnutrition affects between 20% and 50% of patients upon admission, and if prompt measures are not taken to address this issue, patients may experience further deterioration during hospitalization. Even well-nourished patients can become malnourished due to complex metabolic changes occurring in the context of acute inflammation that promote catabolism and disrupt normal nutrient utilization [1]. Certain patient groups, including neurological and elderly patients, face a higher risk of malnutrition due to motor deficits, swallowing disorders, edentulism, age-related issues, challenges in food handling, and inefficient mastication. Other comorbidities, such as diabetes mellitus, hypertension, and chronic cardiovascular and pulmonary diseases, further impact appetite and metabolism, increasing the risk of malnutrition. The hospital diet, because of its modified texture, unpleasant taste, or insufficient caloric and protein content, can reduce the patient’s motivation to eat adequately. All these factors elevate the likelihood of an inadequate diet and the onset of aspiration pneumonia [2].

Malnutrition is associated with a greater risk of mortality, prolonged hospital stays, and increased healthcare costs. However, its impact on clinical outcomes in the intensive care unit (ICU) remains uncertain. Critically ill patients, who are often in a proinflammatory state that worsens their nutritional status, may be particularly susceptible to the adverse effects of malnutrition. Unfortunately, diagnosing malnutrition has proven challenging in earlier studies [3]. For example, serum albumin (ALB) is considered a nutritional indicator but also a marker of disease severity [4]. Additionally, nutritional screening, which assesses the risk of malnutrition, has often been used instead of nutritional assessment, which diagnoses malnutrition, potentially leading to classification errors. Validated tools, such as the Subjective Global Assessment and the Mini Nutritional Assessment, which are recommended by international organizations, are needed to accurately assess the impact of malnutrition in ICUs.

Critically ill patients can develop ICU-acquired weakness (ICUAW), which can persist for a long time after discharge from the ICU. ICUAW results from sepsis, immobilization, and the administration of various drugs, including sedatives, beta-blockers, and corticosteroids. These factors cause metabolic disturbances characterized by intense catabolism, increased protein degradation, and reduced protein synthesis, leading to a rapid loss of skeletal muscle mass [3,4]. It can result in muscle atrophy and critical illness polyneuropathy, prolonged ICU stays, and various complications during the post-hospitalization recovery period if the patient survives [5,6]. Assessing muscle mass upon admission is valuable for identifying patients at nutritional risk, enabling adjustments in nutritional therapy during their ICU stay to reduce the effects of ICUAW. Specifically, reduced or inadequate nutritional intake in the ICU increases the risk of disease-related mortality [5,7,8].

A retrospective study in Japan involving 20,773 critically ill patients on mechanical ventilation and reliant on parenteral nutrition (PN) examined the relationship between energy and amino acid dosing and in-hospital mortality [8]. Patients were categorized based on their daily calorie and amino acid intake from days 4 to 7 of their ICU stay. The findings indicated that inadequate amino acid intake (<0.6 g/kg/day), even with sufficient calorie intake (≥20 kcal/kg/day), was associated with a significantly greater risk of mortality. The authors concluded that critically ill patients dependent on PN may have better outcomes if they receive ≥0.6 g/kg/day of amino acids. Therefore, optimal prescription and adequate nutritional support for critically ill patients have been shown to significantly improve patient outcomes [8].

Jubina et al. examined the impact of nutrition provided in the ICU and during the early recovery phase on physical function at discharge [9]. They evaluated 41 patients treated in the University of Kentucky Post-ICU Recovery Clinic for nutritional intake, gastrointestinal symptoms, and access to food during the ICU and early recovery phases. Patients with no food restriction days had shorter hospital stays, improved physical function, and a reduced risk of ICUAW. Jubina et al. also demonstrated that their study subjects experienced significant weight loss and reported changes in nutritional intake due to early satiety or altered appetite after being discharged from the hospital [9].

Published data demonstrate that nutritional support is crucial during the early recovery phase after critical illness. Unfortunately, ensuring optimal nutritional intake for these patients is challenging and often unsuccessful [10,11,12]. Given these factors, we believe personalizing nutrition in the ICU is essential to enhance the quality of care and outcomes for critically ill patients. Recent guidelines offer practical recommendations for the nutritional care of critically ill patients [9,13,14]. The European Society for Enteral and Parenteral Nutrition (ESPEN) has issued recommendations for the nutritional care of patients in the ICU since 2006. In 2019, it published a comprehensive set of guidelines based on evidence and expert consensus [13]. This 2019 guideline underwent a partial revision in 2023 to streamline the communication of essential messages [14].

The ESPEN and the American Society for Parenteral and Enteral Nutrition (ASPEN) both advise using indirect calorimetry (IC) to assess energy expenditure (EE) in critically ill patients once they are stabilized in the ICU. The recommended energy intake is approximately 70% of the measured EE in the initial phase and progressively increases until it corresponds to the total EE. A protein intake of 0.8 g/kg/day is recommended initially, with a subsequent increase to 1.2 g/kg/day after the patient is stabilized, while remaining mindful of protein restriction in patients with renal dysfunction not undergoing extracorporeal replacement therapy [15,16]. Assessing and identifying deficiencies 5–7 days after ICU admission is recommended for patients at risk of micronutrient and vitamin depletion.

**Figure 1:**
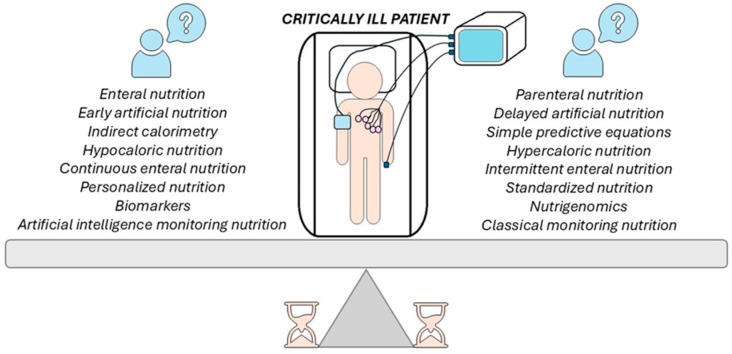
Critical decisions in patients’ nutrition in ICU involve balancing various therapeutic options. On one side of the scale are methods of enteral and parenteral nutrition, the timing of artificial nutrition administration, the use of indirect calorimetry versus simple predictive formulas, and the choice between hypocaloric and hypercaloric nutrition, as well as continuous versus intermittent feeding, personalized versus standardized approaches to nutrition. On the other side are biomarker tests versus nutrigenomics and the use of artificial intelligence for nutrition monitoring versus classical monitoring methods. These dilemmas represent essential challenges in selecting the optimal nutritional treatment for critically ill patients, depending on their clinical conditions and available resources.

Expert recommendations suggest personalizing and adapting nutritional interventions based on the patient’s individual needs, disease-specific factors, and, notably, genetics [17,18]. Personalized nutrition has gradually become a reality with advances in “omics”, including genomics, transcriptomics, proteomics, and microbiomics, as well as in data technology [18,19]. Based on investigative research of the specialized literature, this narrative review summarizes the current ICU nutrition guidelines from ESPEN and ASPEN, along with more recent published evidence. It aims to describe the approach to the central dilemmas currently existing in the nutrition of critically ill patients, with a particular focus on new biotechnological discoveries such as biomarkers, nutrigenomics, and AI. It also addresses the main challenges in implementing personalized nutrition, intending to support ICU physicians who care for vulnerable patients. As illustrated in Figure 1, we posed several questions using articles from the National Center for Biomedical Information—PubMed/Medline journal database published since 1 January 2000. We selected those considered relevant for the subject addressed with updated data. We also found additional articles by examining the reference lists of the previously identified studies.

## 2. Indirect Calorimetry or Basic Predictive Equations

Randomized controlled trials evaluating permissive underfeeding and using supplementary PN to achieve strict calorie control have offered crucial insights into three relevant issues. Firstly, simple weight-based predictive equations (25 kcal/kg/day) provide a clinically helpful approximation of basal EE [20]. Secondly, accurately measuring EE through IC does not seem to enhance outcomes compared to less precise EE estimation. Thirdly, supplying 50–80% of the estimated EE offers clinical benefits comparable to supplying 100% during the early stages of critical illness care [20]. Therefore, it has been argued that the IC estimate is indispensable for determining energy requirements when weight-based equations are inaccurate, such as in patients with anasarca, amputation, severe obesity, or a prolonged hyperinflammatory state. In most other circumstances, routine IC use would not be expected to change the clinical outcomes of early nutritional therapy in the ICU. Therefore, energy and protein requirements are calculated at ICU admission, and EN is initiated within 24–48 h, with PN also initiated when EN is infeasible or insufficient, such as for patients with malnutrition [21].

**Figure 2:**
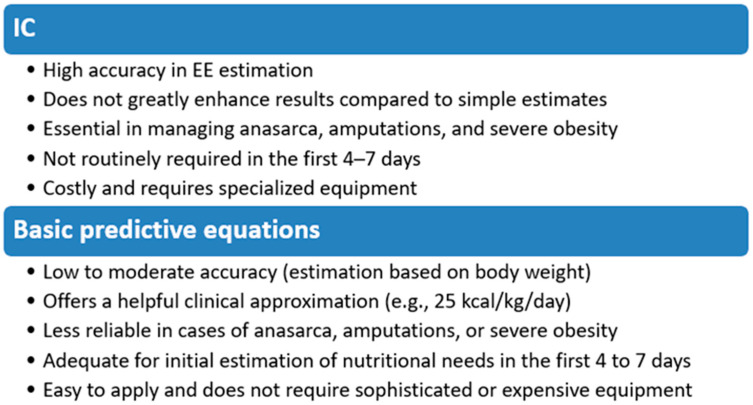
The figure compares two widely used methods for estimating energy requirements in critically ill patients: IC and basic predictive equations. EE—energy expenditure, IC—indirect calorimetry.

The enteral route is recommended over the intestinal route, and low-dose EN can safely begin 48 h after admission, even in the presence of low to moderate vasopressor doses. Notably, the current ESPEN guidelines and expert opinion do not suggest that the nutrition target for critically ill patients should always meet the IC-based EE estimate before days 4–7. High-energy formulas should be limited to patients who cannot tolerate full-volume isocaloric EN or when fluids must be restricted due to associated pathologies. The recommended protein requirement is 0.8 g/kg/day in the early phase of critical illness, increasing to 1.2 g/kg/day in the rehabilitation phase. Vomiting and increased residual gastric volume may indicate gastric issues and intolerance [14,22]. The main characteristics of the two methods for estimating caloric needs are illustrated in Figure 2.

IC remains the gold standard for estimating REE in ICU patients, where possible, offering superior accuracy over predictive formulas, along with adaptability in various clinical contexts and the ability to measure EE dynamically throughout the course of the disease. This reduces errors and makes it an important tool for personalizing nutrition. The usefulness of basic predictive equations persists in units that do not have access to expensive and sophisticated equipment, even though they have significant limitations for critically ill patients. The choice between these methods depends on the socio-economic context, the equipment available in ICU departments, and the presence of adequately qualified medical personnel.

## 3. Enteral or Parenteral Nutrition

Methods for assessing muscle mass, such as ultrasound and computed tomography, also contribute to evaluating nutritional risk and identifying patients requiring close monitoring and appropriate nutrition supplementation [15]. IC is experiencing a “renaissance” in monitoring the resting EE (REE) of critically ill patients. It has become increasingly recommended over the past decade as it allows an objective assessment of basal EE.

The needs of critically ill patients can vary depending on several factors, including the reasons for their admission to the ICU. For example, patients with trauma experience a peak in REE on days 4–5, but their needs remain elevated for the following 9–12 days and even up to 21 days [14,20]. Unfortunately, this method is not routinely practiced in Intensive Care Services, and many hospitals lack sufficient nutritionists to assess risks and provide nutritional recommendations and monitoring. Therefore, a pragmatic approach to nutritional therapy is essential for critically ill patients who remain in the ICU for >48 h. This necessity arises due to particular factors such as mechanical ventilation, continuous renal replacement therapies, extracorporeal oxygenation, sepsis, organ dysfunctions, and multiple organ failure, especially for those who are undernourished for >5 days and at increased risk of malnutrition.

Expert consensus recommends initiating low-dose enteral nutrition (EN) or PN within the first 48 h of admission [14,15]. Both consensus guidelines support EN as the preferred route for nutrient administration, although evidence also indicates that PN can be safely administered without significant risk. Therefore, when early EN is infeasible, providing isocaloric PN is crucial and should be initiated promptly [15].

Adequate nutrition is crucial for both medical and surgical patients, as perioperative malnutrition is associated with impaired healing and compromised immune function [23]. Artificial nutrition is now considered a primary therapeutic intervention for critically ill patients. It aims to prevent malnutrition related to the illness and enhance clinical outcomes. In addition to the timing of initiating artificial nutrition, the administration route is crucial in determining its effectiveness.

EN and PN are both associated with specific risks and complications. PN is easy to administer and tolerated by most patients. In contrast, while less expensive, EN is more challenging to administer and less well tolerated by patients, often resulting in gastrointestinal discomfort and temporary malnutrition [23]. According to a recent systematic review and meta-analysis, critically ill ICU patients who were fed enterally experienced more episodes of vomiting compared to those who were fed parenterally (*p* < 0.01). However, they had fewer anastomotic fistulas (*p* = 0.03) and instances of peritonitis (*p* = 0.03) than the others [24]. The enteral route is considered more physiological, offering both nutritional and non-nutritional benefits, including maintaining intestinal structural and functional integrity and preserving intestinal microbial diversity. Studies have shown that EN reduces the risk of infections and decreases mortality, and initiating it as early as possible is recommended, preferably within 48 h of ICU admission [23,25,26]. PN may be needed for patients who cannot achieve caloric goals with EN. A systematic review found no significant differences in mortality or recovery time between patients who initiated PN early (within the first 48 h) and those who initiated it later (on day 8). Given the infection risks associated with PN, it is recommended to initiate it later, with EN preferred and PN delayed until postoperative day 7, with an ongoing needs assessment [23,27]. PN remains life-saving when EN is impossible. It can be used for patients in ICU settings and home-based patients in some situations. While it has clear nutritional benefits, PN is often associated with infection complications whose etiology is poorly understood. Some theories suggest that its adverse effects result from alterations in intestinal barrier function and an increased proinflammatory status at the mucosal level. These complications arise from enterocyte deprivation and changes in the intestinal microbiome associated with the mucosa [23].

The disadvantages of EN are related to a possible lower digestive tolerance in the acute phase of the disease caused by gastrointestinal dysfunction. Studies have also suggested that survivors of critical illnesses experience weight loss and changes in appetite, including food aversions and persistent gastrointestinal problems, which all affect their nutritional intake after discharge. They have highlighted the need to continue nutritional support after discharge, as adequate nutrition in the post-acute period improves both patients’ short- and long-term outcomes [9,10]. In addition, PN can more effectively ensure the desired nutritional intake, particularly in patients for whom EN is infeasible, such as those with abdominal compartment syndrome or bowel obstruction. However, PN is associated with various complications, the most unpleasant of which are infections. In addition to the previously mentioned theories, other studies support the roles of hyperalimentation and hyperglycemia in PN complications, as well as the translocation of intestinal bacteria due to gut atrophy [28,29,30].

Therefore, previous studies indicate that using EN significantly reduces the incidence of infection complications in critically ill patients and is also more cost effective. Consequently, EN should be the first choice for critically ill patients whenever feasible [31]. The primary distinctions between EN and PN are shown in Table 1.

## 4. Early or Delayed Artificial Nutrition

Early EN offers numerous proven benefits and is systematically recommended by clinical guidelines due to its favorable outcomes [32]. Some published data indicate that delaying the start of nutrition hinders achieving protein energy goals, adversely affecting recovery [33,34], worsening prognosis, and increasing mortality [35,36]. Initiating EN at admission is also associated with a shorter stay and reduced ventilation time than starting EN 24–48 h after admission [33].

In contrast, experimental studies indicate that insufficient autophagy contributes to mitochondrial and organ damage during critical illness. Autophagy inhibition leads to organ dysfunction and increased mortality, whereas autophagy stimulation protects against organ failure and mortality. In the initial stage of sepsis, autophagy serves as a protective response at the pulmonary level. However, in the late stage of sepsis, excessive accumulation of autophagosomes can result in acute lung injury [37,38]. In addition, autophagy was inhibited in the liver and skeletal muscle after the early initiation of PN in experimental critical illness [11].

The timing of initiation and the role of PN—whether as primary, early, or supplementary nutrition—remain controversial. According to a recent systematic review, early PN was associated with a nonsignificant reduction in mortality compared with late PN (RR = 0.95, 95% CI: 0.85–1.06) but with an increased risk of infection (RR = 1.23, 95% CI: 1.08–1.40) [39]. Tolerating macronutrient deficiency for one week in the ICU was associated with better recovery from muscle weakness than receiving early PN. Additionally, macronutrient deficiency did not correlate with early muscle loss during the early phase of critical illness; instead, it facilitated the efficient activation of myofibril autophagy control [40]. Therefore, a significant early deficiency of macronutrients in critically ill patients did not correlate with increased muscle loss but facilitated a more efficient activation of myofibril autophagy quality control and was associated with reduced weakness [41].

During autophagy, autophagosomes form a double membrane that sequesters cytoplasmic components. The fusion of autophagosomes with lysosomes activates lysosomal degradation pathways that degrade damaged organelles and stimulate the innate immune defense against infectious agents [41,42]. Autophagy also serves as a defense mechanism against various invading microbes, and activating autophagy through nutrient deprivation prompts the colocalization of mycobacterial phagosomes with autophagosomes, inhibiting the survival of intracellular bacteria [43,44]. Additionally, some data suggest that early PN may decrease innate immunity by suppressing autophagy, which could increase the risk of infection [42].

While some aspects of the fasting response can be beneficial during critical illness, prolonged starvation has consequences. Studies have shown that early EN is associated with fewer infection complications than delayed initiation in critically ill patients in the ICU. Generally, EN is advised for critically ill patients, with precautions for those experiencing uncontrolled shock, severe hypoxia, acidosis, gastric aspirate > 500 mL/6 h, uncontrolled upper gastrointestinal bleeding, intestinal ischemia, compartment syndrome, or intestinal obstruction, for whom EN is postponed until they are stabilized [45]. The ESPEN and ASPEN guidelines recommend initiating EN within 24–48 h of ICU admission with various nutritional interventions, depending on their clinical factors [13,14,46].

## 5. Hypocaloric Versus Hypercaloric Nutrition

Balanced nutrition, which involves avoiding overeating and undereating, has been associated with improved clinical outcomes in patients in the ICU [47]. In the early phase of critical illness, 50–75% of patients’ energy comes from endogenous glucose production through glycogen mobilization and the mobilization of muscle protein and lipid reserves. This endogenous energy production meets about two-thirds of their energy requirement, necessitating attention to avoid overfeeding [47,48,49].

Controversies remain about the best time to initiate PN in critically ill patients who can meet their caloric needs with EN [21]. Critically ill patients in the ICU often experience significant fluctuations in their metabolic status over a short period, resulting in daily changes to their metabolic state and nutritional needs over their first seven days in the ICU. Patients exhibit considerable endogenous energy production in the early acute phase (days 1–3) and may benefit from hypocaloric support [14,21,50]. Early caloric and protein restriction at disease onset did not increase mortality and was associated with a quicker recovery and fewer complications [50].

In experimental animal studies, fasting-mimicking diets have been associated with increased lifespan and protection against age-related diseases by activating autophagy and enhancing ketogenesis. They also allow for achieving a complete caloric goal while maintaining an improved metabolic profile during complete feeding periods [51,52,53]. Critical illness is accompanied by increased cellular stress and damage, somewhat parallel to normal aging. Therefore, it has been hypothesized that diets that induce fasting might benefit critically ill patients [54,55]. Indeed, it was noted that a 12 h interruption of macronutrient intake was sufficient to trigger a fasting-specific metabolic response in critically ill patients with prolonged hospitalization, as indicated by elevated bilirubin and ketone bodies and reduced insulin requirements and insulin-like growth factor 1 (IGF1) levels [51]. The question arises about whether alternating 12 h feeding and fasting periods could maintain a sustained fasting response and whether it would have clinical implications and benefits [51].

Using IC is recommended to optimize nutritional strategy [55]. Hyperglycemia, an increased insulin requirement, hyperuricemia, an elevated urea/creatinine ratio, and hypertriglyceridemia are suggestive signs of overfeeding, but they are considered nonspecific [47]. In addition, malnutrition can lead to hyperuricemia and an elevated urea/creatinine ratio [51]. Gunst et al. argued that early complete nutritional support does not benefit critically ill patients and may have dose-dependent adverse effects [56]. Therefore, European nutrition guidelines based on recent high-quality evidence have begun to recommend incomplete, less aggressive artificial feeding during the early days of critical illness, advising that complete nutrition should be provided within 3–7 days [13].

## 6. Continuous or Intermittent Enteral Nutrition

EN is recommended for critically ill adult patients, with continuous EN being the most common method of administration; however, the optimal method of administration remains uncertain. EN can be given continuously or intermittently. When constantly administered, the hourly rate remains constant using an electric nutrition pump. When intermittently administered, the total volume is infused over several administrations daily [57]. In healthy individuals, intermittent bolus administration increases mesenteric artery flow, insulin, and peptide YY levels; decreases glucose levels; and enhances protein synthesis [57].

Maintaining glycemic control with reduced glucose variability supports the continuous approach. However, the continuous approach risks hypoglycemia, potentially due to maintaining insulin infusions during short interruptions in EN for bedside procedures or other reasons for different interventions [58]. However, the continuous approach is associated with decreased patient mobility and changes in hormonal secretion over time, potentially leading to long-term metabolic complications, such as hyperglycemia and insulin resistance [59,60].

Skeletal muscle mass is maintained by balancing muscle protein synthesis and breakdown [57]. Insufficient protein intake and starvation lead to muscle loss, whereas protein consumption leads to muscle gain [61]. This muscle protein synthesis effect occurs specifically with “pulse” increases in amino acids, followed by a return to baseline levels 90 min after amino acid intake [61]. The return to basal levels occurs even with a continuous amino acid supply available in plasma and muscle, suggesting that continuous intake does not also stimulate muscle protein synthesis. Therefore, continuous EN or PN could contribute to muscle loss [58].

The intermittent approach involves 4–6 administrations daily using a nutrition pump, syringe, or gravity device [57]. It is considered a more physiological approach than the continuous approach because it provides patients with greater mobility, enhances protein synthesis, and aids in maintaining digestion and regular gastrointestinal secretion hormones [62].

Critically ill patients often present with altered gastrointestinal function due to various mechanisms, such as gastric stasis, gut hypoperfusion, or postoperative ileus. Medical interventions such as sedation, ventilation, and antibiotic use can also lead to gastrointestinal dysfunction [63]. Feeding intolerance presents as vomiting, abdominal distention, aspiration, constipation, or diarrhea, which can lead to malnutrition and greater mortality [64]. In critically ill adults, the continuous approach was associated with lower overall intolerance to nutrition administration, particularly regarding high gastric residuals, gastroesophageal reflux, and pulmonary aspiration. Since the quality of the evidence is low or very low, there is significant uncertainty surrounding this estimate [63]. However, authors such as Qu et al. argued that, based on the available evidence, continuous EN may be more appropriate for patients at greater risk of intolerance to nutrition administration [59]. Considering the limited data available in the literature on these aspects, more high-quality studies are needed to confirm the effects of the two nutrient administration approaches.

## 7. Personalized Versus Standardized Nutrition

Rigorously assessing the energy and protein needs of critically ill patients and adjusting for their specific conditions is essential to optimize nutritional support and mitigate the adverse effects of both undernutrition and overnutrition [65].

Human evolutionary history has been characterized by significant dietary shifts, including the introduction of meat consumption and the thermal preparation of food, which has had a considerable cultural impact and influenced human anatomy, as well as increased the relative size of the brain [66,67,68].

Personalized nutrition, often referred to as nutrigenetics or nutritional genetics, offers insights for customizing nutrition according to an individual’s unique biology [69]. Natural selection influences the frequency of alleles in a population, increasing the prevalence of beneficial ones while decreasing the prevalence of unfavorable ones. Directional selection favors a specific allele, which can become established if the selective pressure persists [69,70,71]. Dietary and environmental changes have generated varying selective pressures, influencing the distribution of alleles across different geographical regions and evolutionary periods. Adaptation to new diets has been based either on new alleles arising through mutations or transferred from other populations or on pre-existing alleles that previously did not provide an adaptive advantage [69,70,72].

Human metabolism is characterized by the simultaneous synthesis and degradation of nutrients to produce energy. Depending on a population’s dietary practices, specific metabolic pathways become predominant due to the emergence of specific genetic adaptations. Most mammals lose their ability to digest lactose after weaning. However, some human populations have maintained this ability into adulthood, likely due to the lactase persistence phenotype, which is particularly common in populations with a long history of pastoralism and who produce and consume milk and dairy products [69,73]. This is an example of positive selection on the lactase (LCT) gene, with genetic variants in the LCT gene identified in various regions of Europe, Africa, and the Middle East [74]. Similarly, amylase 1 (AMY1), an enzyme present in saliva and pancreatic secretions, plays a crucial role in digesting starch. It has been observed that the number of copies of the AMY1 gene is higher in populations with diets rich in starch, such as those that practice agriculture [75]. This evidence demonstrates that specific genetic adaptations have accompanied changes in human diets over time [69].

Genomic studies of human populations have identified genetic adaptations to various diets. Hancock et al. analyzed single nucleotide polymorphisms (SNPs), demonstrating that adaptations to diets rich in roots and tubers were accompanied by changes in genes involved in starch and folate metabolism [76]. Global studies of human genomic variation have revealed significant differences in allele frequencies of common SNPs among populations. These SNPs influence the expression of genes involved in metabolizing essential nutrients, including carbohydrates, fats, proteins, and vitamins [69].

Nutritional genomics aims to develop a rational approach to optimizing nutrition to an individual’s genotype, which defines the relationship between nutrients and human health. Aruoma et al. showed that individuals cannot change their genetics. However, they can eat foods and take supplements appropriate to their genetics to maintain normal cell function and structure. Just as pharmacogenetics examines the genetic causes of individual responses to drug exposure, nutritional genomics examines the interactions between genes and diet, leading to personalized nutritional recommendations [77]. Therefore, an unhealthy diet can be a risk factor for disease.

The human microbiome in the digestive tract represents a personalized genomic signature that can be influenced by various pathologies, organ dysfunction in the ICU, antibiotic treatment, or intestinal tract disorders. Environmental factors such as dietary exposure, including enteral nutrition, can modulate the microbiome [78]. Its importance in producing changes in host physiology, which increases susceptibility to immune dysfunction when the microbiota shifts in critically ill patients, is recognized. Remodeling host–microbiota interactions through personalized nutrition may represent a new disease control and prevention therapeutic approach [79].

Critical illness is often associated with a hypermetabolic state caused by the activation of catabolic hormones that lead to an increased REE compared to a healthy state. Conversely, various iatrogenic factors, including beta-blockers, sedatives, and analgesics, can weaken this response and occasionally trigger a hypometabolic state [49]. Accurate determination of energy requirements is crucial in critically ill patients to prevent the adverse effects of inadequate nutrition. Critically ill patients have a higher EE than healthy individuals, resulting in increased energy needs and a greater risk of malnutrition [80,81]. Undernutrition has been shown to prolong hospital stays, increase the risk of infections and organ failure, prolong the need for mechanical ventilation, and increase mortality. Similarly, overnutrition has been associated with hyperglycemia, hypertriglyceridemia, hepatic steatosis, azotemia, hypercapnia, and mortality [82]. In critically ill patients, nutrition should be personalized and adjusted based on the progression and dynamics of their critical illness. Therefore, IC supporters consider it the gold standard for evaluating the energy needs of such patients [14,80].

## 8. Biomarkers or Nutrigenomics

Personalized nutrition is at the forefront of innovative approaches in nutritional research. This growing field is focused on tailoring nutritional recommendations with unmatched precision by considering diverse individual characteristics [83]. One of the current challenges is identifying new biomarkers that can detect various metabolic dysfunctions early and accurately, predicting the evolution of health status and informing the refinement of nutritional advice for different population groups. Dietary components can alter gene expression, changing protein levels and metabolite composition. Therefore, nutritional interventions can interrupt or restore homeostasis [83]. Consequently, in addition to genetics, microbiome, lifestyle, and nutrition, personalization also explores the complex and highly nuanced field of metabolomics. Metabolomics focuses on small molecules in urine, saliva, blood, and tissues. It can identify metabolic dysfunctions early and form the basis for personalized nutrition management [83,84,85].

**Figure 3:**
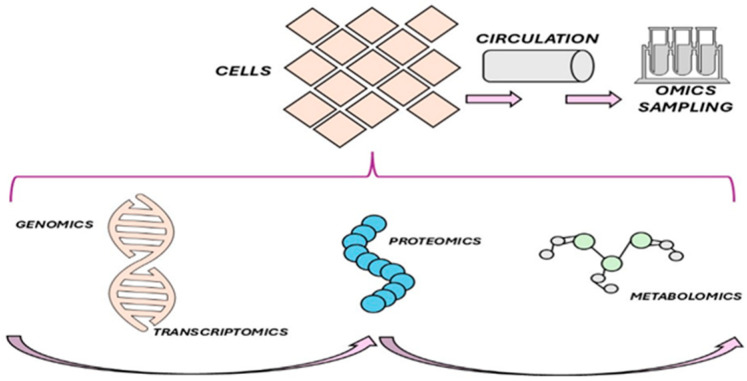
Genomics and metabolomics. The material used for genomics (DNA), transcriptomics (RNA), proteomics (proteins), and metabolomics (metabolites) are extracted from cells collected from an individual. These cells can be obtained from organs, tissues, endothelia, or the circulating blood. Their collection, either by biopsy or, more simply, from the peripheral blood, can provide a window into the omics of specific tissues, cells, or circulating elements.

The metabolome has been increasingly recognized as a valuable resource in recent years, providing unique insights into the intricate interplay of biochemical processes within the body [86] (Figure 3).

Technology developments have enabled studies to analyze global changes associated with health status and nutritional exposure. These technologies offer a more integrated approach to nutrition, considering the interactions between nutrients and the impact of the food matrix on the bioavailability of bioactive compounds [83]. In the past, reductionist approaches concentrated on examining the effects of isolated compounds on specific organs without considering the intricate interactions between nutrients. Today, integrated methods provide a deeper understanding of how foods and nutrients affect health, with metabolomics emerging as a crucial approach in this field [83]. Advances in mass spectrometry and bioinformatics have enabled the measurement of the circulating end products of cellular metabolism [87]. The human metabolome comprises roughly 6500 distinct small-molecule metabolites [88]. Metabolomics is used to identify biological markers of acute and chronic diseases, as well as to assess responses to therapeutic interventions and to elucidate disease mechanisms [89].

While sepsis is a common cause of death, predicting patient outcomes is challenging. Identifying the molecular processes that distinguish patients with sepsis who survive or die could enable more targeted treatments [90]. All critically ill patients show disturbances in metabolic homeostasis. Macronutrients and micronutrients are the primary regulators of metabolite production, exerting their effects through regulating gene expression. The comprehensive profiling of a large number of circulating metabolites in critically ill patients is becoming common [89]. In severe cases, metabolomic studies have consistently demonstrated that alterations in fatty acids, lipids, and tryptophan metabolite pathways are common and associated with disease status and outcomes [89]. Therefore, critical illnesses disrupt metabolic homeostasis, which plays a crucial role in their pathogenesis and subsequent evolution [91].

Proteomic (all proteins in a tissue, cellular compartment, or cell at a given time) and transcriptomic (all RNA molecules in a tissue, cellular compartment, or cell at a given time) analyses of peripheral blood have highlighted systematic changes in patients with critical illnesses, reflected through the milieu of blood metabolites [90]. Langley et al. demonstrated that, in patients with sepsis, the metabolome and proteome differed significantly between those who died and those who survived, particularly regarding fatty acid transport, β-oxidation, gluconeogenesis, and the citric acid cycle [90]. In contrast, regardless of whether they experienced mild or severe sepsis or septic shock, patients who survived did not exhibit significant differences in their metabolome and proteome. An algorithm based on clinical characteristics and measurements of five metabolites accurately predicted patient survival. Therefore, this algorithm could be used to guide the individualized treatment of patients with sepsis [90].

Genetic differences influence individual susceptibility to sepsis and pneumonia. These differences may influence the immune and inflammatory responses during sepsis, resulting in variations in clinical outcomes for patients. An increasing number of genes have been identified as potential candidates for influencing susceptibility to sepsis, indicating a polygenic rather than monogenic effect [92]. Specifically, some of the studied genes that are considered important in both the occurrence and evolution of critical illnesses include mannose-binding lectin (MBL), lipopolysaccharide-binding protein (LBP), bacterial permeability-increasing protein (BPI), CD14 molecule (CD14), toll-like receptor 4 (TLR4), tumor necrosis factor (TNF), lymphotoxin alpha (LTA), interleukin 6 (IL6), interleukin 10 (IL10), plasminogen activator inhibitor 1 (PAI1), and the immunoglobulin G receptors [92].

Metabolomics is increasingly applied in research studies to assess critically ill patients’ responses to nutritional deficiencies and their outcomes management [89]. Nutritional evaluations of critically ill patients can reveal metabolomic changes associated with malnutrition, the dynamic response to nutritional interventions, and the mechanisms underlying the outcomes of nutritional interventions. Integrating metabolomics with transcriptomics and genomics can offer unique insights into how nutrient deficiency and delivery affect cellular homeostasis during critical illness and how homeostasis is restored during recovery [89].

## 9. Artificial Intelligence (AI) or Traditional Methods for Monitoring and Evaluating Artificial Nutrition

Personalized nutrition aims to improve nutritional recommendations by customizing dietary guidelines based on individuals’ unique needs and characteristics. Advances in AI and data technology have enabled the automatic collection and analysis of vast amounts of data, including information from wearable devices that continuously monitor various bodily parameters, providing comprehensive insights into the human body [93]. In the digital age, mobile applications can quickly monitor daily physical activity and calorie intake, correlate these data with user information, and, using AI, provide personalized recommendations on lifestyle and diet [94]. AI is increasingly used due to its ability to analyze, collect, and interpret large volumes of data and apply techniques, including machine learning (ML), which involves using algorithms and statistical methods, particularly in intensive care, where patients require rapid interventions. These techniques can monitor, analyze, and predict patient outcomes, contributing to preventing complications through prompt action [95].

In recent years, AI has begun to play an increasingly important role in health and, thus, nutrition through the ease and security of collecting and analyzing data related to energy and nutrient intake. AI can identify complex correlations and patterns in an individual’s collected data, enabling precise and personalized calculations and predictions compared to classical methods that depend on general estimates [93,94,95]. One study monitored patients who were obese or overweight for one month to develop the AI4Food database. It combined traditional methods with digital techniques and laboratory analyses gathered before and after the intervention to reduce patients’ weight. It included anthropometric measurements obtained every two weeks, questionnaires on lifestyle and diet, and continuous digital measurements over two weeks, successfully highlighting the potential of AI in individualized precision nutrition research [93].

Automated AI-based systems can also identify and manage patients who are malnourished since malnutrition is associated with increased mortality, higher infection rates, and longer hospital stays among older adults. Through AI, food images can be analyzed to distinguish different food components and plate types, estimating the volume of each element before and after consumption and allowing the calculation of the patient’s intake of energy, carbohydrates, proteins, fats, and fatty acids [96]. Its main advantages include automation and real-time analysis, continuous monitoring, reduced human errors, and the integration and analysis of data from multiple sources [95,96]. Traditional methods for data collection and assessment of artificial nutrition can be biased, potentially leading to errors in estimating actual food intake.

Lin et al. conducted a retrospective study to evaluate the impact of caloric deficiency during the first week in the ICU on the outcomes of critically ill patients. By analyzing data from 3544 patients admitted to the ICU for at least 7 days, they found that caloric intake below 60% of the estimated requirements was significantly associated with increased mortality at 28 days (*p* < 0.0001). The risk of death progressively increased in patients who received below 30% of their daily caloric requirements starting on day 5. Conversely, an intake exceeding 80% of their daily caloric requirements starting on day 6 was associated with significantly reduced mortality. This study highlights the importance of adequate nutritional support in the ICU, suggesting potential benefits from intensifying nutritional interventions towards the end of the first week. Its authors developed an electronic system to calculate nutritional intake for critically ill patients, providing 24 h monitoring from 7 a.m. to 7 a.m. This system integrates databases that record data on enteral, parenteral, and intravenous formulas and calories administered for non-nutritional purposes (i.e., glucose and propofol infusions). Through this comprehensive approach, all nutritional parameters, including caloric and protein intake, can be automatically calculated daily [97].

Exploring the use of data-driven technologies in various areas of nutrition, including applications for critically ill patients, could provide a more comprehensive perspective and a deeper understanding. Consequently, AI offers numerous advantages in data collection, analysis, personalized recommendations, continuous nutrition monitoring, and integration of multiple data sources. However, combining AI methods and traditional practices may be the optimal solution for accurate and reliable results.

## 10. Multidisciplinary and Multiprofessional Collaborations in the Nutritional Therapy of Critically Ill Patients

The nutrition management of critically ill patients involves multiple medical disciplines, as well as administrative and political factors. However, the critical care nutrition approach, which involves collaboration among clinical nutritionists, ICU nurses, ICU doctors, clinical pharmacists, and radiologists, is not sufficiently comprehensive to ensure practical cooperation between multidisciplinary teams in many countries, including China [98]. Structured, multidisciplinary nutritional management processes can also guide clinical practice. As noted by Shunxia et al. and supported by evidence, they may be used in the nutritional management of critically ill patients, leading to effective improvements in nutritional indicators, shortening ICU stays, and facilitating patient recovery [98].

Due to the diversity and severity of critically ill patients and their varying nutritional statuses, it is challenging for a single discipline to implement a nutritional support program that meets all patients’ needs. Pathology, age, various treatments, EN administration, analgesic administration, and mechanical ventilation can all contribute to the development of EN-associated diarrhea or constipation, as well as reflux, which can be exacerbated by increasing the EN rate and volume, leading to complications and requiring different multidisciplinary approaches. Disease severity can also cause ICU staff to overlook nutritional support [98].

**Figure 4:**
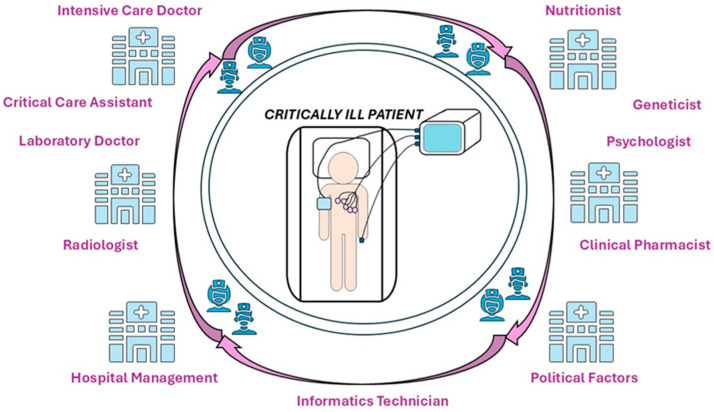
The figure illustrates the complexity of nutritional care for critically ill patients in the ICU, highlighting the multidisciplinary and integrated nature of a medical intervention. The focus is on the critically ill patient, connected to multiple monitoring and life support systems, around whom many specialists (ICU medical team, support specialists) and various technical and decision-making factors revolve. Nutritional decision making in intensive care does not belong exclusively to a single specialist; rather, it results from collaboration among various fields within an organized framework, supported by technology and institutional regulations. The ultimate objective is to provide safe, efficient, and adapted nutrition to the critically ill patient.

Diagnostic and treatment guidelines, supported by a multidisciplinary team, have become increasingly vital in the modern medical model. It is essential to gradually apply these guidelines across all areas of medicine and throughout the patient’s treatment process [98,99,100]. The nutritional management of critically ill patients is essential. It should involve a multidisciplinary team and various professional collaborations, including ICU staff, clinical nutritionists, clinical pharmacists, radiology technicians, laboratory physicians, geneticists, IT specialists, and factors related to political and institutional management (Figure 4) [98,100]. Nutritional health policies and institutional management play a critical role in the success of nutritional therapy for critically ill patients since they influence access to resources, the formation of multidisciplinary teams, and the implementation of standardized protocols [101,102]. Furthermore, efficient management at the institutional level can ensure the proper allocation of resources, continuous monitoring of patients’ nutritional status, and effective coordination among the various specialties involved. Their collaboration is vital for therapeutic success [98].

## 11. Challenges and Barriers to Implementing Personalized Nutrition

The current costs of advanced diagnostic tools, such as genomics, proteomics, or metabolomics, make them infeasible in the ICU, particularly in settings with limited financial resources. Nutrigenomics encompasses the interactions between genes and diet, focusing on how genes influence the body’s response to nutrition and how nutrition affects the body’s response to defective genes. Advances in understanding the complex interactions between genotype, lifestyle, diet, and environment can lead to personalized nutritional recommendations that aim to reduce disease risks and enhance patient outcomes and well-being [77,101,103]. Given that dietary components can alter gene expression, practitioners must recognize that diet influences health and disease, depending on an individual’s genetic makeup. While individuals cannot change their genetics, they can consume foods appropriate to their genetic predispositions and take appropriate supplements to address genetic imbalances, promoting normal cell function and structure [77,104]. In order to implement genomics in artificial nutrition, healthcare professionals must be able to evaluate the genetic tests offered, demonstrate extensive knowledge in the field, and provide appropriate recommendations based on their understanding of the evidence and its clinical applicability. These skills are essential to maximize the value extracted from nutrigenetic tests ethically and responsibly [77].

In order to implement a personalized nutritional intervention, it is crucial to develop predictive models that monitor the body’s responses to food in real time through data integration at various levels and advanced ML techniques. Using AI and medical databases enables the creation of predictive models that consider individual variability, providing personalized nutritional recommendations [105]. In addition, AI offers numerous advantages and possibilities in healthcare systems, particularly in patient nutrition. Portable, non-invasive medical devices can help monitor patients’ vital signs, including electrocardiography, skin temperature, blood pressure, pulse, and peripheral oxygen saturation, promptly alerting medical personnel to any detected abnormalities. This technology also helps to reduce the workflow, allowing medical personnel to focus on patients with special needs or unstable conditions [106,107]. Other challenges of personalized nutrition include the limitations of reductionist approaches, the need for robust computer infrastructure, the lack of standardization in data from electronic health records, and incomplete data. Advanced technologies such as AI and ML, data standardization, and the development of efficient methods to predict missing data are required to address these barriers [105].

Combined with AI, technical advances in the medical industry have transformed the healthcare sector. Consequently, individuals concerned about their health are becoming increasingly aware of the market offerings and the availability of devices that incorporate AI, monitor various parameters, and utilize them on a large scale [107].

Currently, the use of AI in ICU patient nutrition is relatively limited and prohibitive for some units due to the high costs involved. Nonetheless, this new technology can integrate multiple data points that reflect the dynamic needs of patients and identify those at high risk of inadequate nutrition that could compromise their health prognosis. By identifying these patients, prompt interventions, including nutritional support, are linked to a better prognosis, reduced hospitalization, and increased survival rates, ultimately helping to decrease overall costs. Therefore, such investments have the potential to pay for themselves through improved patient outcomes [108].

The importance of personalized nutrition in the ICU cannot be overstated. We have reached a time when clinical nutrition, EE, and energy intake can be accurately measured in the ICU, a capability that was not available until the implementation of the discussed discoveries. Indeed, the hope is that new AI technologies and centralized nutrition platforms will ultimately lead to improved clinical and functional outcomes in the long term.

## 12. From Theory to Practice: Implementing Personalized Nutrition in the ICU

The heterogeneity of ICU patient cases is striking. Phenotypic characteristics, pre-existing nutritional status, the type and severity of associated pathologies, and the associated inflammatory response influence nutrient requirements and nutritional treatment tolerance. While traditional nutrition guidelines are applicable, they often do not cover all these aspects, particularly the dynamic changes in critically ill patients.

The exaggerated immune response and systemic inflammation that accompany infection lead to extensive tissue damage and organ failure. Critically ill patients exhibit complex and evolving metabolic profiles, often marked by profound catabolism [109]. Intensive care nutrition has recently shifted from a “one-size-fits-all” approach to a more personalized strategy to optimize metabolic support and improve prognosis.

Traditional nutritional approaches do not consider the distinct needs of patients based on body composition and metabolic stress that arise from the pathology leading to their admission to the ICU. In contrast, phenotyping (BMI, sex, age, medical history) and monitoring unintentional caloric intake (from dextrose solutions, propofol) address the patient’s specific characteristics, including body composition and the micro- and macronutrient requirements at various stages of the disease [110,111]. The use of specific metabolic biomarkers and the identification of various mechanisms involved in the individualized pathology of patients admitted to the ICU can help develop personalized nutritional plans [110]. Personalized nutrition in the ICU integrates patient-specific data (metabolic needs, genetic characteristics, microbiome composition, and response to therapy) with individually tailored nutritional interventions [109,112]. However, determining variations in energy expenditure and endogenous energy production is often challenging at the bedside [113].

As previously discussed, early feeding can induce overeating, insulin resistance, and hyperglycemia. Protein and micronutrient (vitamins, trace elements) administration using endotyping (intracellular biomarkers) and phenotyping is a personalized approach, as protein requirements may vary depending on these factors [110]. In some cases, high protein intake may be linked to increased mortality due to metabolic intolerance. Assessing anabolic resistance to protein synthesis, autophagy, and the urea/creatinine ratio (a marker of metabolic utilization of ingested proteins), along with personalized protein dosing and monitoring muscle mass through ultrasound, can improve prognosis in the ICU by preventing excessive or insufficient protein intake [114,115,116].

However, implementing a personalized nutrition plan in the ICU faces numerous obstacles, such as the lack of institutional protocols regulating interdisciplinary collaborations among ICU doctors, nurses, pharmacists, and especially nutritionists within the critical care team. Most often, the attending physicians establish the nutritional plan, leading to significant limitations in personalizing nutritional therapy [117]. Law 256 of 2015, with additions made in 2024, regulates the activities and practices related to the exercise of the dietetic profession and the establishment, organization, and operation of the Romanian College of Dietitians. However, it does not explicitly regulate the involvement of dietitians in ICU teams, and too few clinics have employed a dietitian. Unfortunately, in our country, there is generally a minimal number of dietitians employed in hospitals, and they contribute to the creation of generally adopted menus for specific pathologies such as diabetes mellitus, hypertension, gastric ulcer, and diverticulitis, without creating personalized menus [118].

Under these conditions, nutritional care in Romania is deficient. This refers to the coordinated approach of health professionals providing fluids and food to individuals with varying needs within collaborative nutritional care. Moreover, studies show that in other intensive care centers, patients consistently receive insufficient calories and proteins, particularly during the first week of critical illness, with only 3–5% of ICU wards reaching adequate levels of calories and proteins [119,120]. In contrast, other countries, such as Australia, have tested a Systematized, Interdisciplinary Malnutrition Pathway for imPLEmentation and Evaluation (SIMPLE) in hospitals. This study is based on an interdisciplinary collaboration model aimed at improving the nutritional management of malnutrition across six Queensland hospitals [121].

Due to insufficient human resources and the prioritization of emergency treatment, assessing patients’ nutritional status is often conducted late. However, it should occur within the first 24 to 48 h and at regular intervals thereafter [122]. The lack of necessary equipment for accurately monitoring body composition and logistics, such as computerized programs for calculating nutritional needs and intakes, contributes to a more generalized approach [123,124].

It is estimated that, in 2016, approximately 8.6 million deaths were recorded due to ineffective and delayed medical care in low- and middle-income countries [125]. Treating patients in the ICU requires expensive equipment, highly specialized staff, and complex medical procedures, all of which contribute to rising costs. Studies show that the mortality rate in intensive care units (ICUs) ranges from 8% to 33%, and after discharge from the ICU, mortality in regular hospital wards can reach between 11% and 64% [126]. Intensive care is an expensive medical specialty that requires advanced technology and well-trained staff, so it is often considered a low priority by public health authorities in resource-limited countries.

Paradoxically, the costs of medicines and equipment can be higher in low- and middle-income countries due to import duties and a lack of domestic production. Additionally, insufficient numbers of well-trained medical personnel and inadequate measures to control hospital-acquired infections favor the occurrence of complications, altering outcomes and further increasing costs [125,127].

The burden of preventable deaths, along with patients who survive with significant sequelae, also represents losses and expenses for state economies. This is why public health authorities in low- and middle-income countries should invest in and encourage the development of ICU units, as the cost-effectiveness balance is favorable for the care of critically ill patients, according to a study conducted in a recently established medical ICU in Sarajevo, Bosnia and Herzegovina. Survival rates and median quality of life indexes support this premise [126].

Implementing a personalized nutrition plan for ICU patients is essential. Overcoming the barriers to practical implementation requires issuing clear legislative regulations so hospitals can develop effective protocols and strategies for interdisciplinary collaboration. This includes integrating the nutritionist into the ICU team while securing adequate national health insurance funding. Although the ASPEN and ESPEN guidelines include clear protocols, not all hospitals apply or adapt these recommendations, and in some cases, the Romanian guidelines are not sufficiently updated. Continuing medical education courses, programs, computerized data integration, and clear legislative regulations are vital for achieving these goals.

## 13. Conclusions

Implementing personalized nutrition in the ICU is a complex and difficult challenge, but it can potentially improve outcomes and the progression of critically ill patients. Although there have been obvious technological advances, barriers still persist related to increased costs, inadequate infrastructure, and insufficient specialized personnel, especially dietitians, complicating the implementation of these strategies in clinical practice settings. However, utilizing advanced technologies like AI alongside genomic and metabolomic analyses could provide innovative solutions for dynamic monitoring and appropriate adaptation of nutrition interventions.

Future ESPEN/ASPEN guidelines must integrate AI algorithms that, combined with metabolomics, provide insights into the extent and nature of metabolic changes and allow for rapid nutritional decision making. Technological innovations and AI play a promising role in personalizing nutrition for critically ill patients.

Therefore, the priorities should include developing standardized protocols for interdisciplinary collaboration, investing significantly in precise monitoring technologies, and continuously educating and adapting medical professionals.

## Figures and Tables

**Table 1:** The table compares EN and PN, two essential methods of nutritional support for critically ill patients, based on several clinical criteria; EN—enteral nutrition, PN—parenteral nutrition.

Criteria	EN	PN
Management and expenses	It is more challenging to manage, but it is less expensive	It is easy to administer, but it is more expensive
Tolerance	Reduced digestive tolerance during the acute phase	Generally, well tolerated
Common complications	Nausea and vomiting, temporary malnutrition, a reduction in fistulas, and instances of peritonitis	Infections, elevated blood sugar levels, and intestinal atrophy
Nutritional benefits	Preserves the integrity of the intestines	There are no benefits for gut health
Effect on infections and mortality	Reduces the risk of infections and death	There is an increased risk of infections, but no clear differences in mortality rates
The best time to begin	In the first 48 h	Day 7 after surgery
Indications, long-term use	The digestive tract is functioning properly; post-discharge monitoring might be necessary	Intestinal obstruction, can be sometimes managed at home, depending on certain conditions
Efficiency of caloric intake, recommendations	Sometimes it is insufficient, choose as the first option if possible	Offers a more efficient way to meet energy needs and is an alternative when EN is unavailable

## Data Availability

Data are available based on request from the corresponding author. The data are not publicly available due to time limitations.

## Abbreviations

The following abbreviations are used in this manuscript:

AI	Artificial intelligence
ALB	Serum albumin
AMY1	Amylase 1
ASPEN	American Society for Parenteral and Enteral Nutrition
BPI	Bacterial permeability-increasing protein
CD14	CD14 molecule
DNA	Deoxyribonucleic acid
EE	Energy expenditure
EN	Enteral nutrition
ESPEN	The European Society for Enteral and Parenteral Nutrition
IC	Indirect calorimetry
ICUAW	ICU-acquired weakness
ICUs	Intensive care units
IGF1	Insulin-like growth factor 1
IL6	Interleukin 6
IL10	Interleukin 10
LCT	Lactase
LBP	Lipopolysaccharide-binding protein
LTA	Lymphotoxin alpha
ML	Machine learning
MBL	Mannose-binding lectin
PAI1	Plasminogen activator inhibitor 1
PN	Parenteral nutrition
REE	Resting EE
RNA	Ribonucleic acid
SNPs	Single nucleotide polymorphisms
TLR4	Toll-like receptor 4
TNF	Tumor necrosis factor
